# Supplementary figures and images for: Boundary vector cells in the goldfish central telencephalon encode spatial information
Source: PLoS Biol. 2023 Apr 25;21(4):e3001747. doi: 10.1371/journal.pbio.3001747 (PMC10128963; doi:10.1371/journal.pbio.3001747)

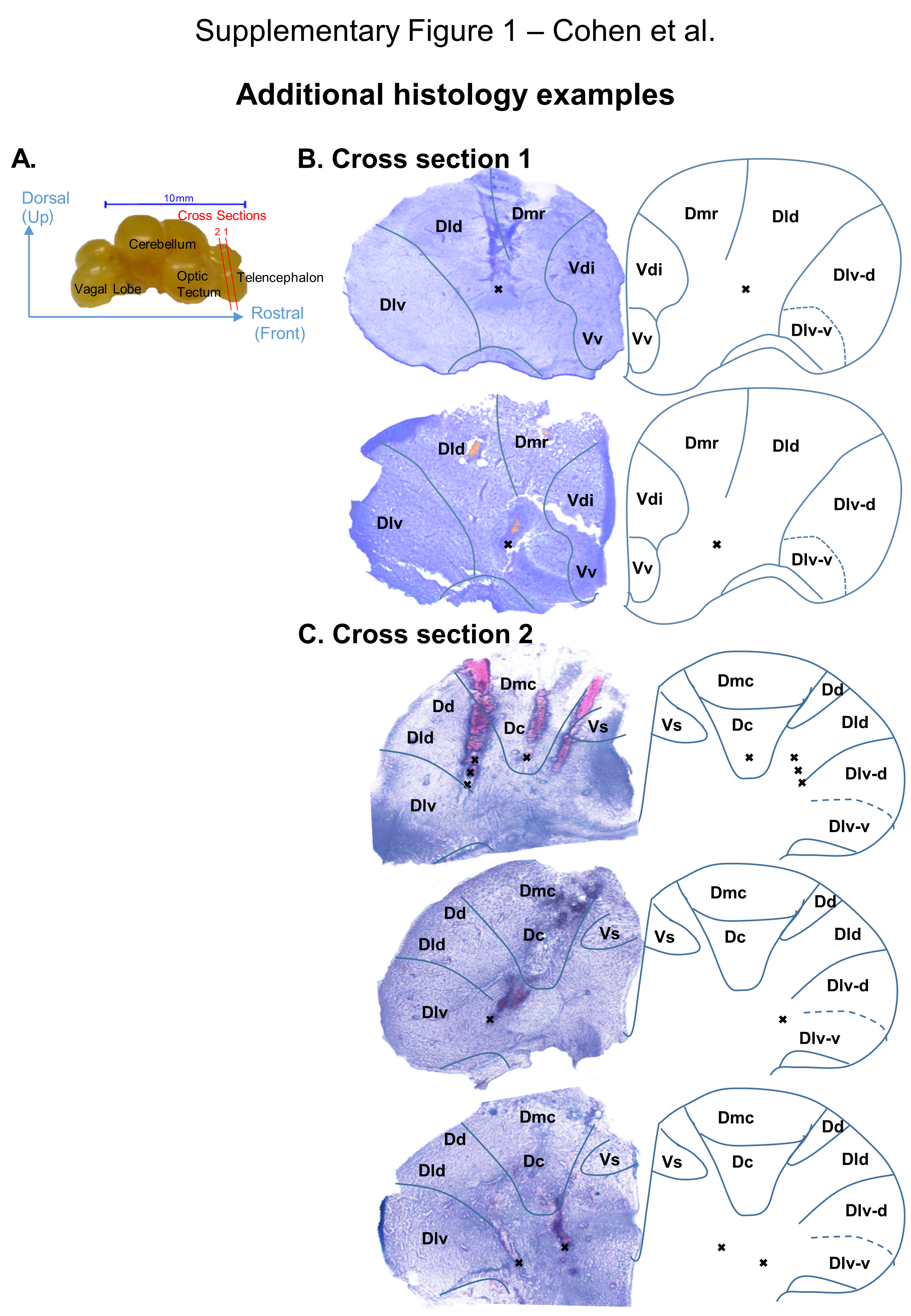

Supplement: S1 Fig — (A) Fish brain structures. Red cross: Sections 1 and 2 correspond to the brain sections presented in panels B and C, respectively. (B, C) Examples of recording sites in the goldfish central telencephalon and the corresponding brain region (right panel, anatomical diagram based on [40]). Black x’s show location where the boundary vector cells were recorded. (TIF) [file pbio.3001747.s001.TIF]

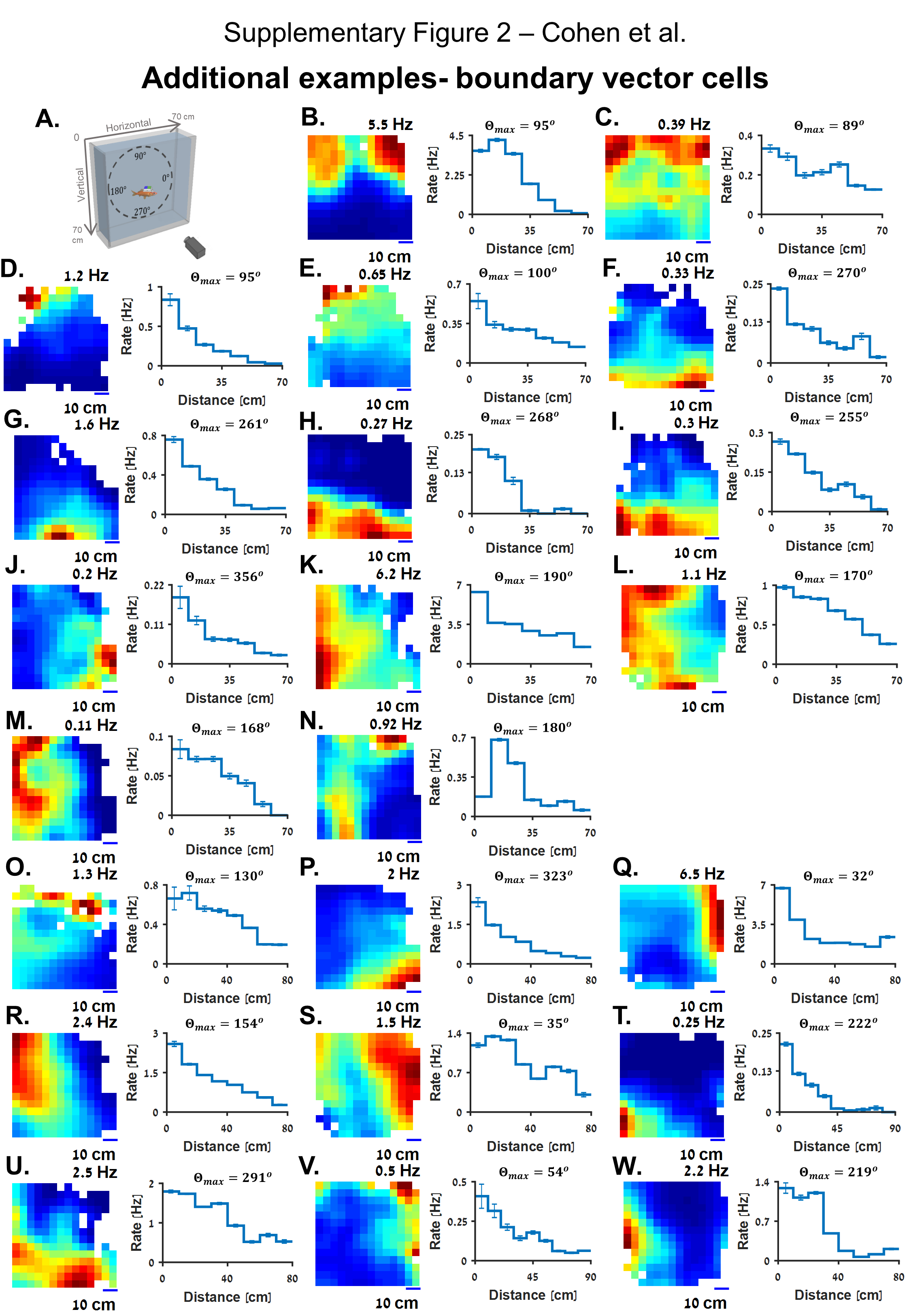

Supplement: S2 Fig — (A) Schematic overview of the experimental setup. Dashed circle presents the Θmax axis. We used camera-east as zero with an anti-clockwise progression. (B-W) Boundary vector cells examples. For each cell presented are the firing rate map (left panels) color-coded from dark blue (zero) to dark red (maximal firing rate, indicated) and the tuning curves (mean ± standard deviation, right panels) of firing rate to the distance of the fish from the preferred boundary. Preferred direction (Θmax) is indicated. (B-I) Vertical tuning examples (Θmax = 90o±15o or 270o±15o). (J-N) Horizontal tuning examples (Θmax = ±15o or 180o±15o). (O-W) Other tuning examples. The underlying data supporting panels B-W in this figure can be found in a file named SuppFig 2_data.mat (see Data Availability). (TIF) [file pbio.3001747.s002.TIF]

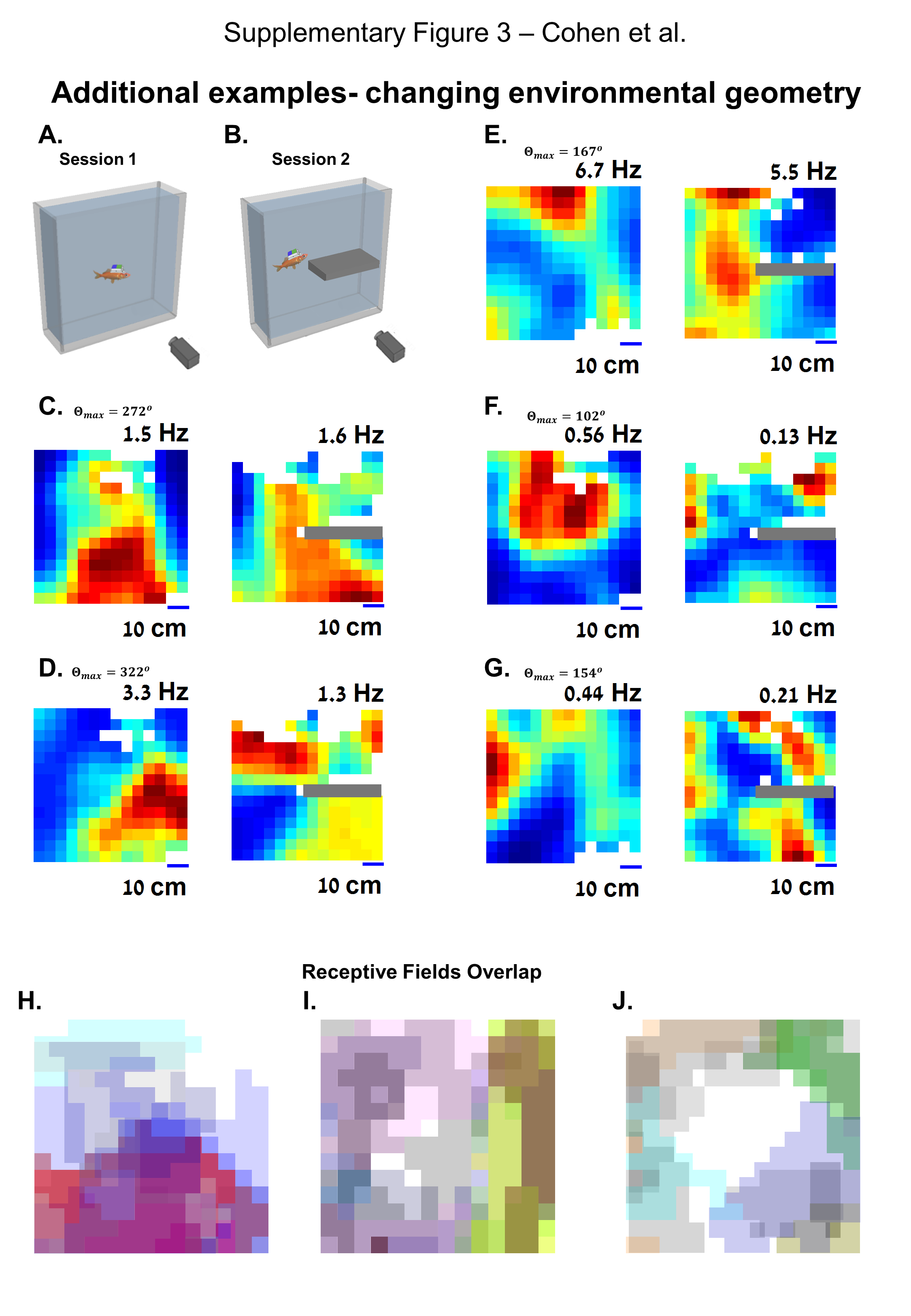

Supplement: S3 Fig — (A, B) The cells presented in panels C-G were recorded first in the main experimental water tank (A). Then, before the second recording session, a shelf was inserted into the water tank to modulate the geometry of the environment while the fish was blindfolded. (C-G) Examples of boundary vector cell rate maps before (left panels) and after (right panels) the geometric change in the environment. Maps are color coded from zero (dark blue) to the maximal firing rate of each cell (dark red, indicated). (H-J) Overlap of the entire 35 boundary vector cells. Each color represents the bins in space in which one cell was firing at a rate of at least half of its maximal rate. The population covers the entire water tank. For assessment only, the population was divided into 3 different panels: (H) cells with a vertical tuning direction, (I) cells with a horizontal tuning direction, and (J) cells with diagonal tuning direction. The underlying data supporting panels C-J can be found in a file named SuppFig 3_data.mat (see Data Availability). (TIF) [file pbio.3001747.s003.TIF]

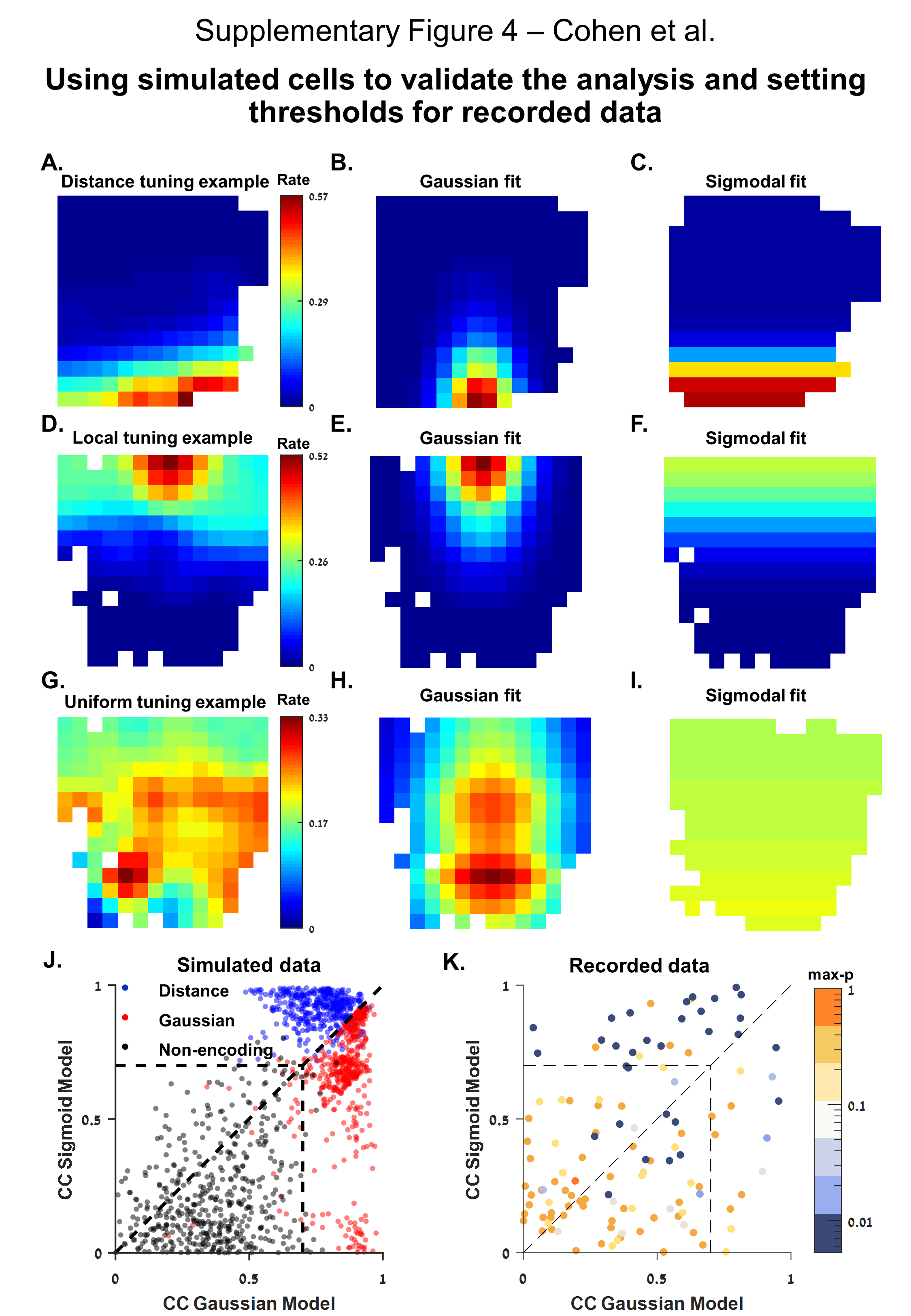

Supplement: S4 Fig — Three groups of cells (500 cells in each group) were simulated with different tuning properties in space. For each group, two models were tested to characterize the resulting firing pattern. (A) Example of a simulated rate map with a distance tuning pattern. Same color bar was used for the rate maps in panels B and C. (B) A rate map fitted to the rate map in A using 2D Gaussian tuning. (C) A rate map fitted to the rate map in A using a sigmoid tuning. (D-F) Another simulated example with a local tuning pattern near a boundary in the environment. (G-I) Another simulated example with a uniformly distributed firing pattern in space. (J) Three groups of 500 firing patterns were simulated, and correlation coefficients were calculated for each fitting method (see Materials and methods). The three groups—distance encoding, Gaussian encoding, and non-encoding—could be classified using boundaries of 0.7 and above or below the identity line. (K) Correlation coefficients between recorded data rate maps and the rate maps generated by a Gaussian model and a sigmoid model (see Materials and methods). Results suggest that the boundary vector cells can be better described with a sigmoid firing pattern in space rather than a Gaussian firing pattern. Thresholds (dashed lines) were derived from the simulated dataset (panel J). The color bar on the right-hand side of panel K spans the population max-p values (see Materials and methods) on a logarithmic scale such that the blue dots represent the strongest spatially modulated cells. The underlying data supporting all panels in this figure can be found in a file named SuppFig 4_data.mat (see Data Availability). (TIF) [file pbio.3001747.s004.TIF]

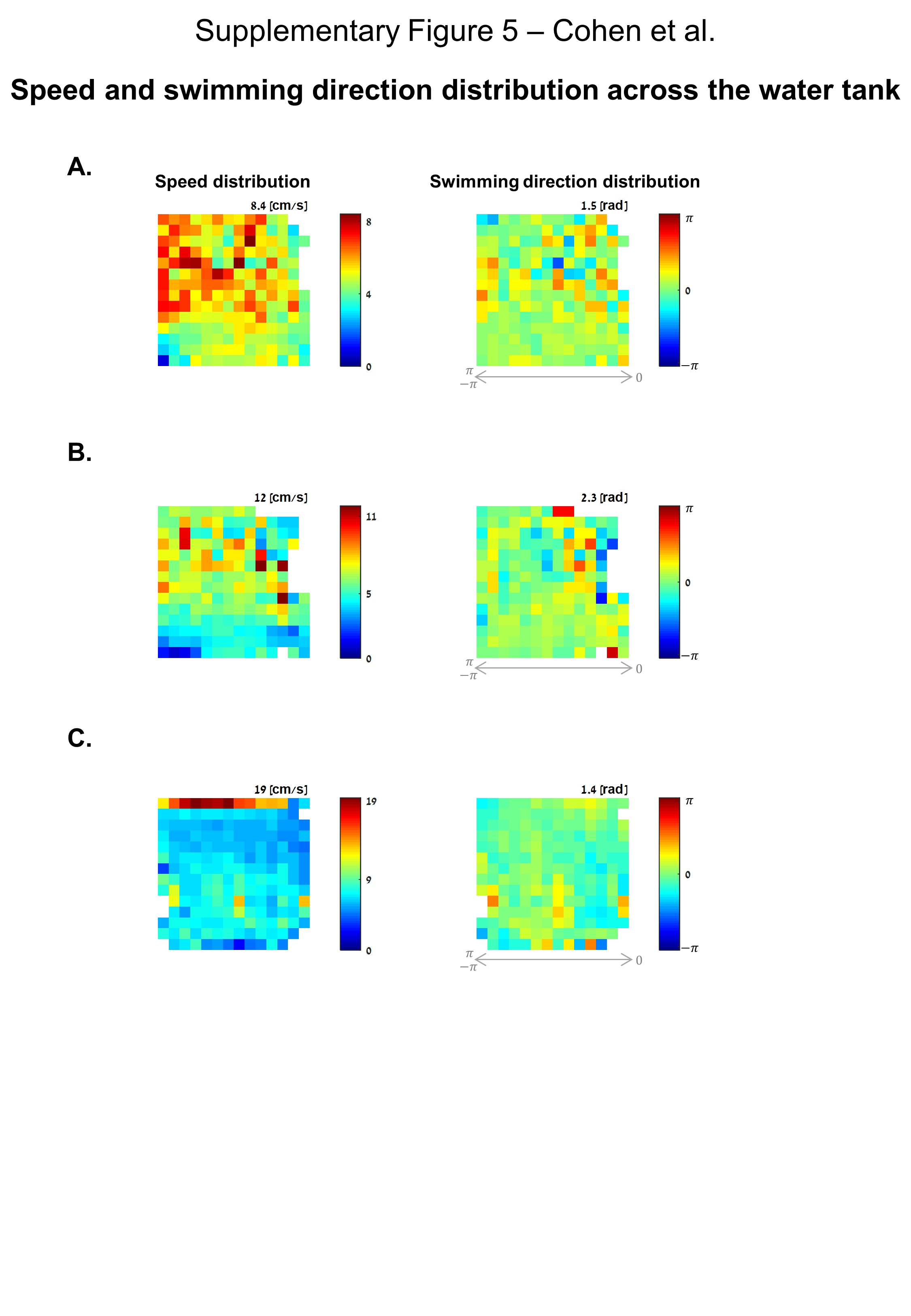

Supplement: S5 Fig — Examples of swimming speed and allocentric swimming direction distributions of the boundary vector cells presented in Fig 2. For each cell, a swimming speed map (left panels) is presented, color coded from dark blue (zero) to dark red (maximal swimming speed, indicated on the top right side of each panel). Also shown are the allocentric swimming direction map (right panels), color coded from dark blue (−pi) to dark red (+pi). No clear patterns emerged. Examples correspond to the cells presented in (A) Fig 2A–2D and 2M–2P, (B) Fig 2E–2H, and (C) Fig 2I–2L. The underlying data supporting all panels in this figure can be found in a file named S5 Fig_data.mat (see Data Availability). (TIF) [file pbio.3001747.s005.TIF]

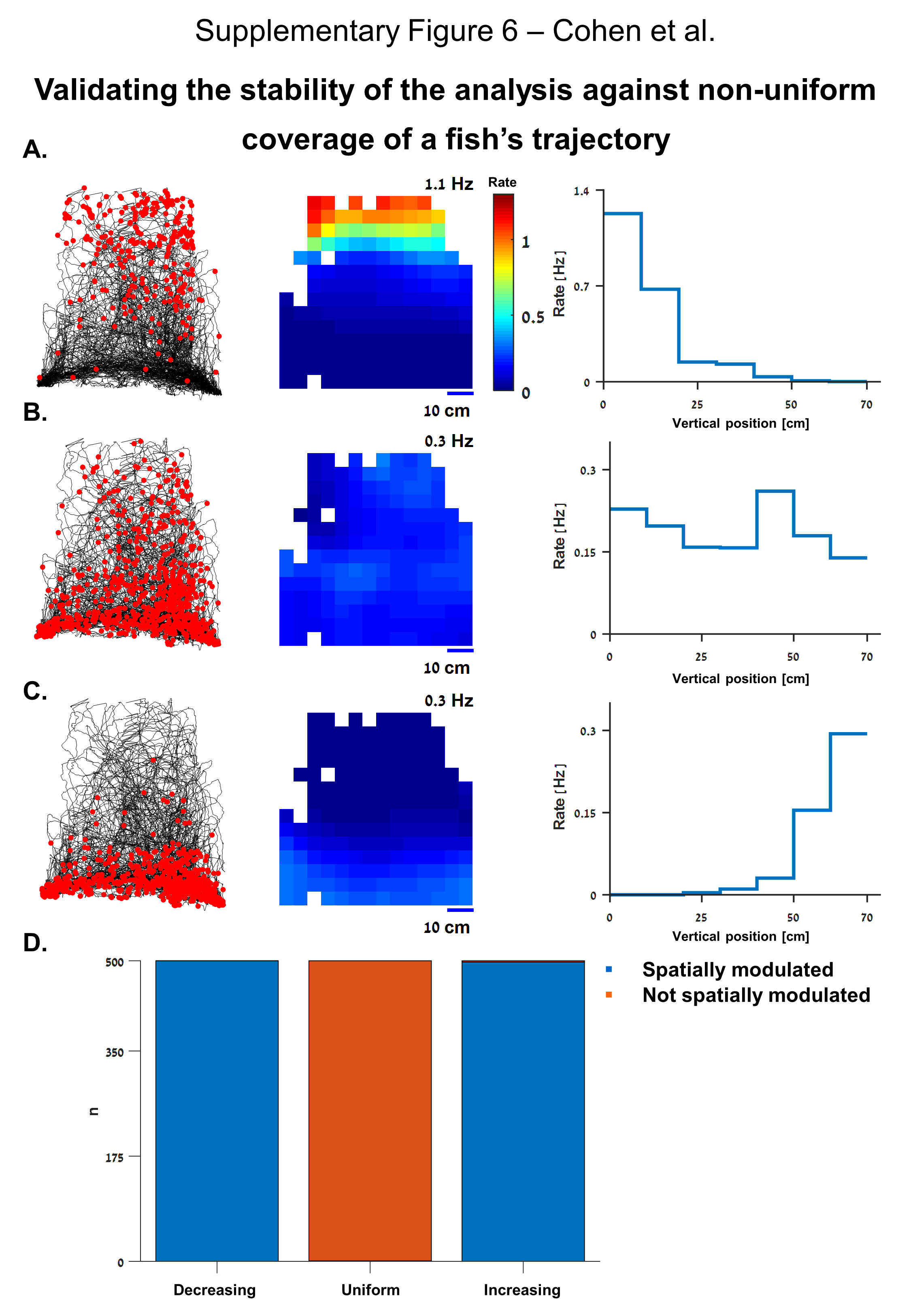

Supplement: S6 Fig — We used three tuning curves of firing rate to position in the vertical axis as recorded from three different fish to simulate firing patterns in a real trajectory (panel A, left panel, black curve), which partially covered the experimental water tank. This trajectory corresponds to the cell presented in S2D Fig. For each tuning curve, we simulated 500 spike trains. (A-C) Examples of spiking patterns (red dots, left panels) over the swimming trajectory (black curves) together with a color-coded occupancy-corrected heatmap (middle panels, same color bar as in panel A) and the tuning curves used to simulate them (right panels). The tuning curves either gradually decreased (A), were quasi-uniformly distributed in space (B), or gradually increased (C) with position along the vertical axis of the water tank. (D) Simulation results. Each simulated spike train was then tested to determine whether it crossed the threshold for a spatially modulated cell (see Materials and methods). Out of the 1,500 spike trains, 1,497 were classified correctly (false negative rate <0.002). The underlying data supporting all panels in this figure can be found in a file named S6 Fig_data.mat (see Data Availability). (TIF) [file pbio.3001747.s006.TIF]

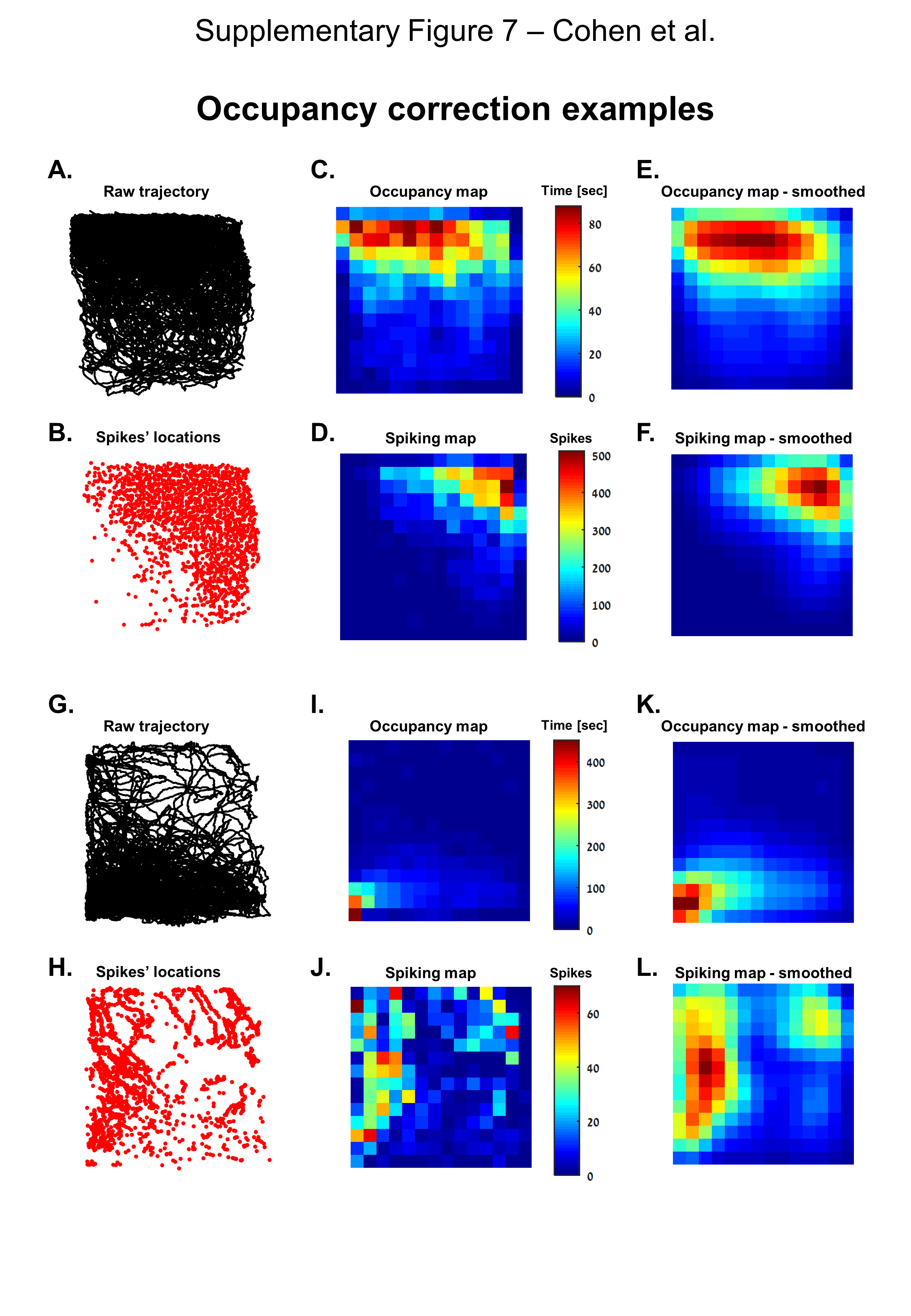

Supplement: S7 Fig — (A) Trajectory and (B) an example of spiking locations (correspond to the cell shown in Fig 2I). (C, D) The fish’s trajectory and spike positions were binned using a 5 cm × 5 cm grid to generate an occupancy map (panel C) and a spike per bin map (panel D). (E-F) The maps were then smoothed using a 2D Gaussian (σ = 20 cm) to obtain auxiliary maps of occupancy per bin (panel E) and the spike count per bin (panel F), respectively. A bin-by-bin division of these maps yielded the occupancy corrected heatmap as shown in Fig 2J. (G-L) Another example, corresponding to the cell shown in S2B Fig. The underlying data supporting all panels in this figure can be found in a file named S7 Fig_data.mat (see Data Availability). (TIF) [file pbio.3001747.s007.TIF]

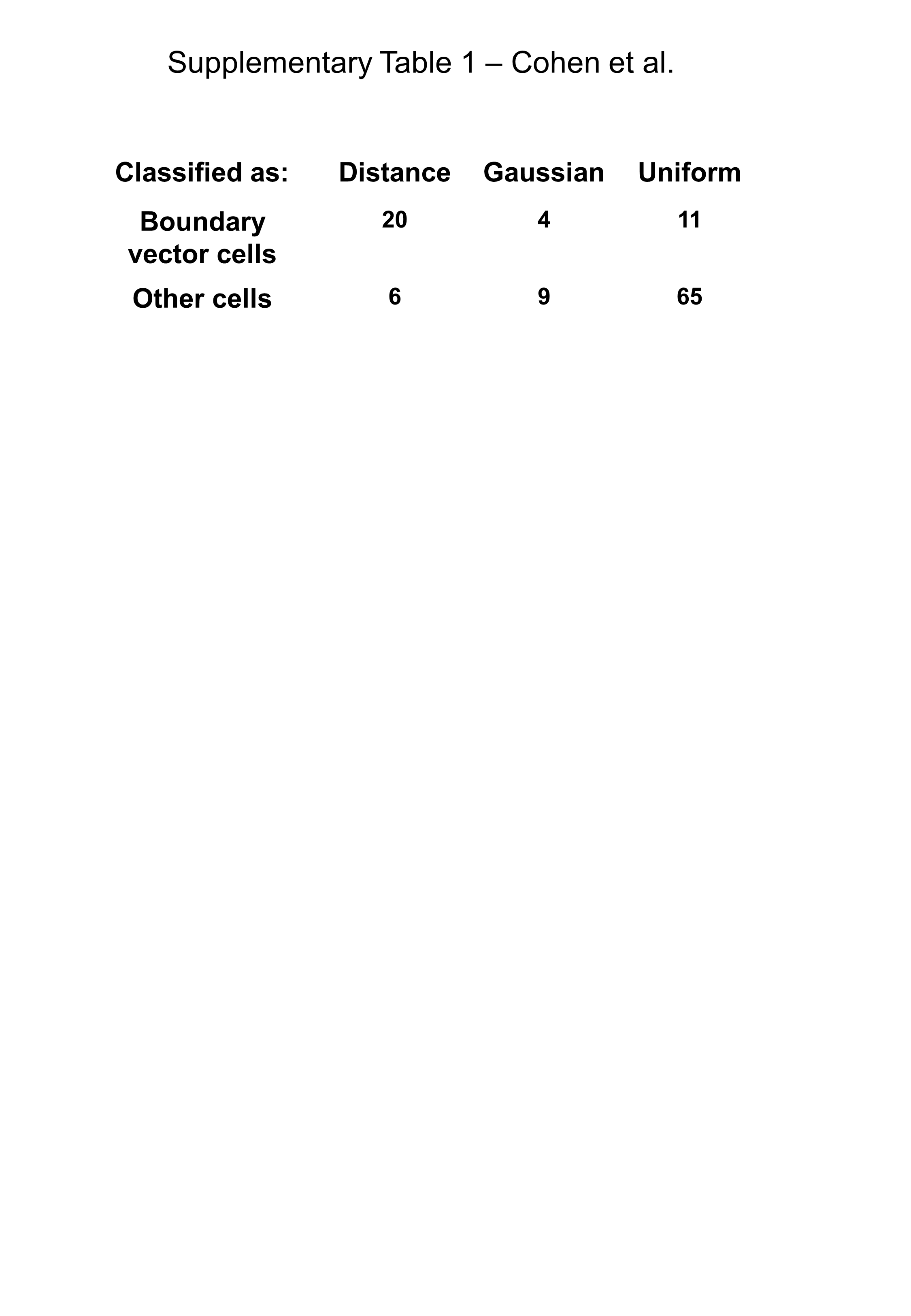

Supplement: S1 Table — In correspondence with the results presented in S4K Fig. (TIF) [file pbio.3001747.s008.TIF]

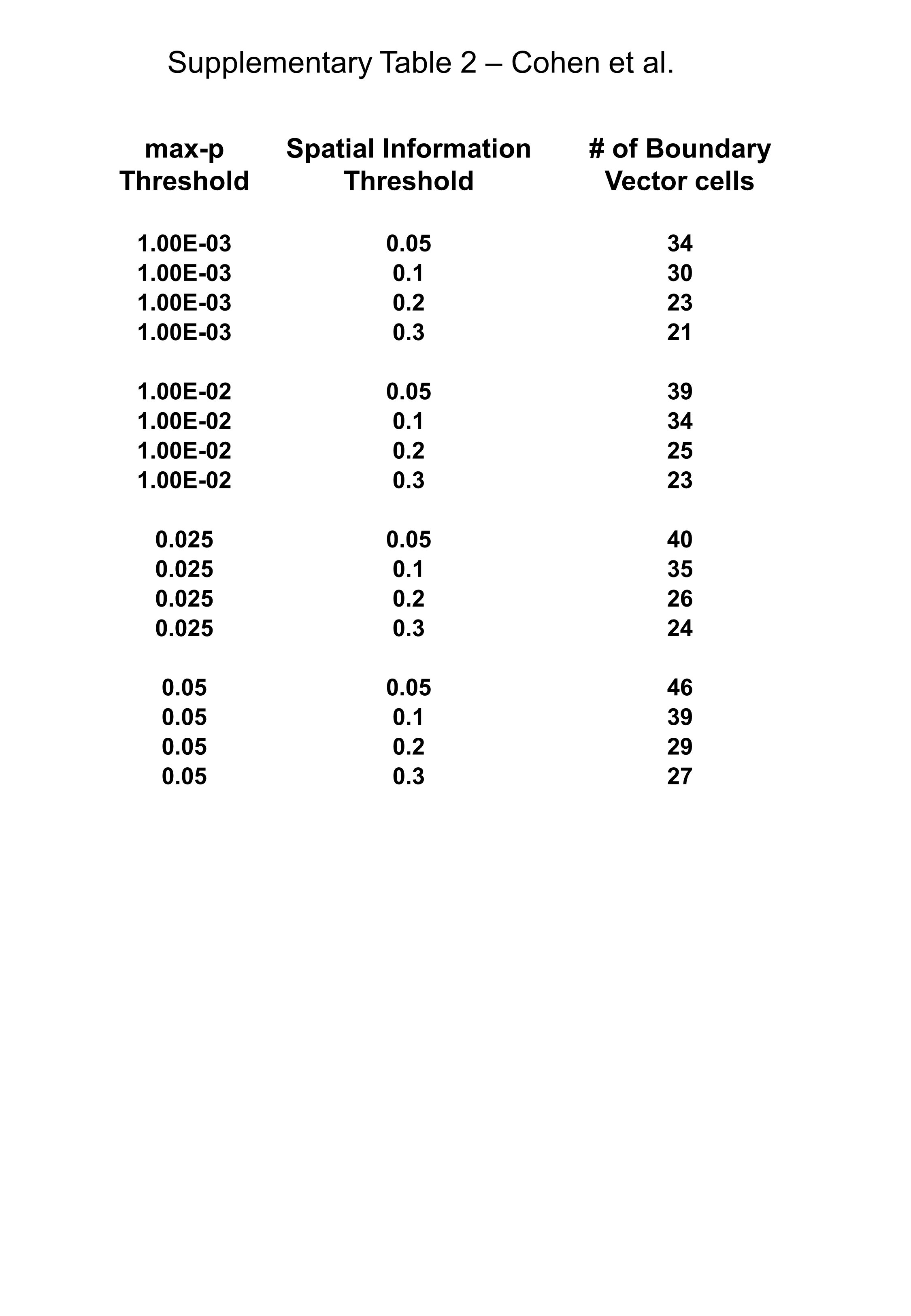

Supplement: S2 Table — Different max-p values and spatial information thresholds (see Materials and methods) were tested to show that the prevalence of boundary vector cells in the population is not strongly affected by the chosen thresholds. (TIF) [file pbio.3001747.s009.TIF]
