# Supplementary material for: Boundary vector cells in the goldfish central telencephalon encode spatial information
Source: PLoS Biol. 2023 Apr 25;21(4):e3001747. doi: 10.1371/journal.pbio.3001747 (PMC10128963; doi:10.1371/journal.pbio.3001747)
Supplement: Attachment — Submitted filename: Cohen2022_BVC Goldfish_Response to reviewers.docx [file pbio.3001747.s013.docx]

**General response to the referees’ comments**

We are grateful to the referees for their thoughtful comments and the time they devoted to reading our manuscript.

A joint comment from all referees was concerned about terminology used to describe the cell we found, and which we termed 'axial encoding cells'. The referees pointed out that the cell types we found in the goldfish have many similarities with the boundary vector cells previously described in the mammalian brain. They also pointed out that it might be better avoiding using new terminology and instead using existing terminology used in the mammalian literature.

We agree with the referees and decide to change the terminology. Specifically, cells that were previously labeled ‘axial encoding’ are now called ‘boundary vector cells', as suggested. We of course describe differences between the boundary vector cells found in the goldfish central telencephalon and the cells found in the rat's subiculum.

The remainder of the responses deals point-by-point with the comments made by each referee and explains the revisions made to address them. Referees’ comments are depicted in black font, our responses are in red font.

**Reviewer #1**:

This is a very original study on navigational neurons in fish, which I read with great interest and pleasure. This is to say, the manuscript is well written describing up-to- date methods and ambitious, as well as demanding experiments. The authors clearly establish a new concept of 3D space representation in the telencephalon of teleosts, in this study the goldfish. Instead of the well described place cells, grid cells boundary-vector cells, and head direction cells in mammals and birds they found neurons with firing patterns that gradually increased or decreased along spatial axes distributed in all directions in 3D space. These results were obtained from freely swimming fish in a water tank by wireless tetrode recordings. The data are convincing, and interpretations are modest and made safe by the statistics applied.

This a study perfectly suited for PLOS Biology, and I recommend acceptance after minor revision Specific comments:

I am irritated by the figures showing the swim paths superimposed with the action potentials and the transformation into heat maps, although it is done correctly. Sometimes it is counterintuitive to compare the firing pattern on the swim path to the heat map because the red area in the heat map do not always correspond to the number of action potentials or the other way round many spikes on the swim path lead to a blue area in the heat map. Therefore, it would be helpful and clarifying in the legend of figs. 2, to clearly state the consequences (or necessity) of occupancy correction. I suggest having both swimming path with spikes and heat map only in figure 2 and in the subsequent figures only show the heat maps. The information in the two figures is redundant anyhow.

We agree with the referee, and the figures were revised accordingly. As suggested, we now only show trajectories in Figure 2. Furthermore, we added insets showing the spikes and trajectory separately, as requested by referee 2 (see Figure 2).

The occupancy correction process is standard and essential in this type of analysis and widespread in the literature. It helps to avoid artifacts due to inhomogeneous coverage of the arena by the animal, as animals do not sample their surroundings uniformly. This process is described in detail in *Material and Methods* (lines 407-419), in Supplementary Figure 7 and in the caption of figure 2. We also ensured our analysis was valid even when the tank was not covered homogenously (Supplementary Figure 6).

Fig 2 D: I see no red or blue arrows, or do you refer to the vertical lines?

The red arrows were difficult to see because of their size and they have been corrected to make them more visible.

Fig. 3 there is some tuning in the orange curves corresponding to the orthogonal axis. Wouldn't that imply a broad gaussian activity distribution. I like the test sigmoid versus gaussian. But a better fit to sigmoid does not exclude also a gaussian distribution. Please elaborate on this point.

We thank the referee for this comment. The referee is correct and a better fit to sigmoid does not exclude also a gaussian distribution.

The focus of our analysis is to rule out that the cells we recorded were a subset of narrowly activated place cells that happened to be centered near boundaries rather than boundary vector cells. As the referee commented, a 2D very broad Gaussian distribution is similar to a sigmoid distribution. Thus, we cannot exclude a Gaussian distribution by a better fit to a sigmoid. Nevertheless, our analysis does show that the cells are not typical place cells because they do not fit a narrowly-distributed Gaussian. To distinguish more accurately between a sigmoid and a broadly tuned Gaussian we would need additional experiments in larger water tanks. All the above is now mentioned in the relevant sections in *Materials and Methods* (lines 508-556) and in *Results* (lines 242-261).

It should be mentioned that in the new context of boundary vector cells, this analysis is of secondary importance, and therefore was maintained as part of the supplementary material in the revised manuscript (Supplementary Figure 4).

Discussion
Since the transformation from a sigmoid to a gaussian function mathematically is straightforward I would invite some speculation how evolution could have changed the axis coding to place cells.

In the new context of boundary vector cells, we discuss the differences between the cells found in fish and the ones found in mammals with respect to their habitat and physiology (see *Discussion*, lines 303-324). While the questions about the mechanism of evolution of place encoding cells are intriguing, we believe that we need to deepen our understanding of place encoding in the fish brain before we can start answering this question.

The distinction between the boundary vector cells (rats) and axis cells (fish) is difficult and at least in some examples to me seems impossible, e. g. heat maps in fig 2F, J and N.

We agree with the referee about this point which is in-line with the other referees’ comments on this issue. We now indeed refer to the cells in the paper as boundary vector cells.

**Reviewer #2**:

The authors present a follow-up study to a 2018 paper by the same group. The main claim of the paper is that different from the previous paper that observed border cells, the cells in the current papers present spatial axes with different orientations.

While the authors have performed a large number of difficult experiments, and their approach of investigating place fields in non-standard species, and particularly in 3D is certainly very interesting and worthwhile, the current presentation of the data as well as the experiments makes it difficult for the reader to follow the author's conclusions. I hope the following comments will contribute to clarify their findings.

Major comments:

-With regards to previous experiments: a slightly larger tank, vertical instead of horizontal was used here. In how far does the visual surrounding around the tank impact the place fields? Could they just look different than in the 2018 paper due to different lab surroundings, i.e. visual cues?

More generally, how was the fish able to get oriented in the environment? What were the visual cues around the tank? Does rotating the tank where the fish is modify the tuning of the cells?

We thank the referee for raising these points. Previous behavioral studies have shown that goldfish use multiple cues such as in-tank visual cues, out-of-tank visual cues, tank geometry and the hydrostatic pressure gradient.

The fish in our experiments can use the existing visual cues in the setup. Specifically, the walls on 3 sides of the tank (one horizontal side and the two short sides) were covered and blocked the visual cues from the room. The water tank faced a corner of a room, and the only visual cues from the outside came from the uncovered horizontal side where a camera system together with a computer are placed. The tank was open at the top, so the ceiling was visible to the fish as well. The water level (and the resulting hydrostatic pressure) was kept constant during all the experiments. To become familiar with the experimental water tank, the fish trained in it with feeding prior to the implant (*Results*, lines 62-78). Details about the tank and the familiarization have been added to the *Materials and Methods* section (lines 373-383).

In our case, rotation of vertical tank is not straight forward as in horizontal tank. In horizontal tank, we can completely rotate all visual cues. In a vertical tank, we can rotate the visual cues, but we cannot rotate the hydrostatic cue. This makes a fundamental difference between the two environments. While the issue of depth encoding is important, we have decided to leave it for future study as it requires a whole different experimental design and controls.

Furthermore, the cells we observed in this study had different firing patterns than the edge cells in the previous paper. In the current study we observed wide fields, stretching over a large portion of the tank, that were activated by a single or even partial boundary. On the contrary, edge cells of the previous study were constantly activated by all 4 walls in the environment and were tuned to shorter distances from the boundaries. Specifically, none of the 119 single cells recorded in this study had passed these criteria for edge encoding.

All of this is now further clarified in the text (*Results*, lines 87-94, and 181-198, and *Discussion*, lines 290-302).

-The main argument that these are not border cells seems to come from figure 4, where changing the border leads to changing fields.

However, based on all the data shown, it seems that the fields are generally strongest at the borders or in the corners. A maybe more conclusive experiment could be to insert a diagonal wall (at 45 degrees) into the tank. If these are not border cells but indeed encode spatial axes, you would not expect to find a cell strongly firing along the diagonal.

We felt the referee’s suggested control experiment was an important one, and we decided to conduct it. The actual new experiment we did was slightly different than the one proposed by the referee, to enable it to correspond to the new definition of these neurons as boundary vector cells. In addition, the new experiment we adopted is common in the field.

In the new experiment we inserted a horizontal boundary within the arena. The new results show the effect of an additional boundary on the firing patterns of boundary vector cells (see Figure 5 and Supplementary Figure 3).

The main argument that these are not border cells (as in the mammalian form) comes from the major difference in the field they cover. Whereas border cells are active close to the borders, the cells presented in this manuscript encoded a large portion of the water tank (67% on average, Figure 6A) with a gradually decreasing firing pattern with distance from the boundaries. This is also in line with the suggested idea of boundary vector cells in rats [Lever 2009] that border cells might be a subset of boundary vector cells with a smaller field size. They were also very different from goldfish edge cells for the reasons mentioned in the previous comment.

Altogether, we believe the recorded cell population can be best described as boundary vector cells.

-Also, to get a better idea of the variety of the recorded cells, I would suggest to show all the recorded data in the SI (with 30 cells this could fit on 2 pages).

We agree with the referee’s suggestion. The entire boundary vector cell population (aside from the cells presented in Figures 2, 5 and 7) is now presented in Supplementary Figures 2-3.

I would also suggest to plot an overlap of all the place cells, to see how they cover the aquarium, that is to see the spatial covering of the tank. Showing examples that are not in the corners or at the edges would make the argument more convincing that these are not border cells (as stated around line 120).

The suggested overlap plot was added to Supplementary Figure 3 H-J (divided into three panels for a better visual assessment).

An example cell not firing near the border, which is more like the boundary vector cells with a large receptive field shown in [Lever 2009] than like a typical mammalian border cell, is also presented (Supplementary Figure 2M).

-Please plot the trajectories and corresponding firing rates in Fig. 2 and all other similar plots in separate figures, in many cases the red dots of the firing rate make it impossible to assess the spatial distribution. Please also show some examples in supplementary information how the densities of space and firing rate were calculated and how the calculation of the final place fields was done. Discussing how walls, corners, or center of the tan are represented, might also help following the argument that these cells encode spatial axes.

We thank the referee for this comment. We added insets showing the spikes and trajectory separately to make this clearer (Figure 2). Furthermore, we added Supplementary Figure 7 showing the occupancy correction method as suggested. The receptive field was measured as the width of the sigmoid with the least square error from the data's rate map (Figure 6A, see also *Materials and Methods,* lines 508-556).

We now refer to these cells as boundary vector cells and not as encoding spatial axes.

Minor comments:

-Fig 3: C, F, I and L : please show some error band for these rate profiles.

Error bars were added.

- Can the position of the fish at maximum tuning be decoded from neural activity?

We thank the referee for this question. Although we agree that there is a clear gradual connection between firing rate and position, a decoding analysis for position is complicated and will be examined in future work.

-All references are mixed up, so I was not able to follow in how far the literature is cited accurately

We apologize for the inconvenience caused in the initial submission. This has now been corrected.

-The authors repeatedly mention 3D space, for example in the abstract, but the analysis is only for 2D and the third shorter axis is ignored. It would therefore be more accurate to just refer to 2D. Overall it seems that the experiments would be considerably more conclusive if a 3D tank could be used.

We thank the referee for this comment. We made sure all 3D are in the right context now.

-Line 105: Typo, "a geometric change [was] induced in the environment."

Corrected.

-Line 125: please also include these data in the suggest figure showing all recorded place fields

All the boundary vector cells in the paper are shown in the suggested overlap (Supplementary Figure 3 H-J).

-Fig 6G, missing y-axis range

Corrected (now figure 7E).

-The introduction claims that the place cell is the most prominent one among the space encoding cells, I would change the wording on that, since many people might disagree with that statement.

The referee is correct. This has been revised.

-Why was velocity not analyzed as in previous work? Does the firing rate depend on the direction in which the fish swims?

We thank the referee for this comment. In the current setup, the fish's head direction had a limited range relative to the environment as they tend to stay horizontal most of the time. Therefore, we could not analyze "velocity cells" as in the previous work.

Nevertheless, we added an analysis of allocentric swimming direction, and we now show that this effects the activity of the boundary vector cell population (see Figure 4). The lack of correlation between swimming direction and position and between swimming speed and position is also presented (Supplementary Figure 4).

-How many cells were recorded at the same time and if more than one, did they ever differ in spatial selectivity?

In each surgery, 3-4 tetrodes were implanted. The largest number for a single experiment was 12 single cells (8 recorded on a single tetrode, 2 of them were classified as boundary vector cells), but this is of course an exception. The largest number of boundary vector cells recorded in the same experiment was 5 (two on one tetrode and three more on another tetrode). The three recorded boundary vector cells had 3 different preferred directions (see Figure 2 A-B and M-N and Supplementary Figure 2A). This information was added to *Results*, lines 62-86.

-Supplementary Fig 3 seems to be one of the main arguments that these cells represent spatial axes. However, that fitting of a 1d sigmoid works better than a Gaussian is not convincing and likely indicates a problem with the fitting routine. There is clearly 2d information in these distributions. The 2d gaussian fit should converge to a distribution with one very wide axis, if indeed that were the best fit, so basically approaching a sigmoid, but this seems not to happen, so likely the parameters on the Gaussian were set wrongly or too limiting.

We thank the referee for this comment. The referee is correct and a better fit to sigmoid does not exclude also a gaussian distribution.

The focus of our analysis is to rule out that the cells we recorded were a subset of narrowly activated place cells that happened to be centered near boundaries rather than boundary vector cells. As the referee commented, a 2D very broad Gaussian distribution is similar to a sigmoid distribution. Thus, we cannot exclude a Gaussian distribution by a better fit to a sigmoid. Nevertheless, our analysis does show that the cells are not typical place cells because they do not fit a narrowly-distributed Gaussian. To distinguish more accurately between a sigmoid and a broadly tuned Gaussian we would need additional experiments in larger water tanks. All the above is now mentioned in the relevant sections in *Materials and Methods* (lines 508-556) and in *Results* (lines 242-261).

It should be mentioned that in the new context of boundary vector cells, this analysis is of secondary importance, and therefore was maintained as part of the supplementary material in the revised manuscript (Supplementary Figure 4).

-In figure 2 M and J, that represent diagonally tuned cells, also the trajectories of the fish don't seem to cover the corners; the trajectories themselves seem slightly diagonal, which might lead to a confound in the analysis. Why is that? Are the corners not accessible?

The entire water tank was accessible. In many cases the fish did not cover the entire environment homogenously. This was true for all fish even before the implant surgery. Therefore, we conducted a simulation in which we show our analysis is not sensitive to inhomogeneous coverage of the water tank (Supplementary Figure 6). The possible confound is also the reason why the occupancy correction was used (see examples in Supplementary Figure 7).

-More generally, is there a correlation between the spatial distribution of the trajectories and the resulting axis of the place fields?

There can be a difference between the occupancy and the spiking activity. Specifically, in Supplementary Figure 7, which shows the method of occupancy correction we present two such examples. In addition, as mentioned above, sometimes multiple cells with different spatial selectivity were recorded in the same experiment and hence were not correlated with the trajectory.

-Was the information along the narrow axis recorded? It would be interesting to know whether cells fire more at the borders or whether firing is equally distributed between the two walls, which would provide another strong argument for or against border cells.

We thank the referee for this comment. We do not have information about the position of the fish along the narrow axis. The size of this axis was small, and therefore even if we had an additional camera placed alongside the water tank, this information would be difficult to analyze. Future work will include experiments in a full 3D setup and in natural size environments.

-What is the significance of beta oscillations in the authors view? It does not become clear from the discussion why this finding was presented or what it signifies in the authors view.

We thank the referee for this comment. The oscillatory properties in the mammalian hippocampal formation are subject to extensive research. The relationship between oscillations and spatial encoding is an important aspect of space encoding. As far as we know, there is no evidence in any other clade for the relationship between beta oscillations and space encoding. The uniqueness of the results presented here is the finding of beta oscillations associated with position encoding in fish. This points to the relationship of oscillations and spatial representation in non-mammalian vertebrates.

This is important because this study is part of a comparative approach to the well-studied mammalian hippocampus. Therefore, it provides another perspective on the evolutionary basis of these differences and for the importance of rhythmic oscillations for space encoding. This has now been added to the text (*Discussion*, lines 310-318).

**Reviewer #3**:

There are several exciting aspects of the report, which combine to produce a very interesting dataset. These include: 1) the clarity and robustness of the spatial signal, analysed using different spatial measures. 2) The discovery of beta oscillatory modulation, particularly in the spatial cells, in this region (central telencephalon), potentially speaking to the importance of oscillatory modulation in spatial coding. To my knowledge, there has been no report of any kind reporting beta oscillations in fish, so the novelty is clear.  3) Importantly, because fish are moving in a 3-D world, the examinable set of firing responses can now be extended from rodent literature to include, like the bats, those involving the Z dimension. Indeed, exactly as those familiar with the Boundary vector cell (BVC) model would predict, the BVCs in this report include BVCs with directional tuning preferences involving the down/gravity direction or the up/anti-gravity direction. Thus, a cell which fires at the bottom of the tank, but with a right-wall (or 'camera-east') bias, is a boundary vector cell which is tuned to fire at a short distance from a boundary in the down-east direction. Thus, excitingly, there is species continuity in boundary vector cells across fish and mammals. (The authors are oddly reluctant to apply what seems like the obvious BVC category label to these cells, which I mention further below.)

The paper offers a step-like increase in understanding vertebrate spatial representations, providing a vital piece of the jigsaw in terms of species-comparative knowledge, and it is surely likely to appeal to a wide readership. The authors' previous paper in Scientific Reports was more modest in terms of discovery but has been well cited. I think this reflects the strong interest and appetite for comparative approaches to spatial cognition (insects, fish, birds, rodents, bats). The present findings certainly deliver on that front. Accordingly, all taken together, I enthusiastically recommend publication in PLOS Biology.

There are, however, important revisions to make to the paper. In general, many details (some methods, but mostly analytical characterisations of the cell population) are missing from what might turn out to be a seminal report in an interdisciplinary journal. These details are crucial for others to replicate, contextualise, compare, and build upon the findings. Moreover, as I point out below at various points, I don't think the authors' conceptual framing of this population of spatial cells in terms of 'axial coding' is accurate or helpful, and in fact is somewhat misleading. The authors have discovered boundary vector cells in the dorsal telencephalon of goldfish, but don't seem to want to say this. My sense is that this is accidental and reflects understandable specialism in the fish rather than rodent literature.

Title: 'Representations of 3D space'

It is certainly true that the fish were freely moving in 3D space, but the authors do not describe representations of 3D space, and this must be removed from the title and any mentions in main text. In their analysis, what they call the length dimension (20 cm), has in effect been collapsed, perfectly understandably, onto a plane.

We agree with the referee. We made sure that all "2D" and "3D" noted in the manuscript are now in the correct context.

More detailed characterization

These details are crucial for others to replicate, contextualise, compare, and build upon the findings.

A) Results

I would expect to see the following characterisations

Towards the early part of the results

 i) The absolute number of cells that are 'modulated by the fish's position' (p.5), alluding to how this has been defined. If the authors employ two definitions for different analyses (>0.1 bits/spike p.23 I think is the second definition), specify which one the initial "~30%" figure (p.5) refers to. It seems simplest to move the bottom paragraph on page 11 (describing the 31 passing the combined 3 criteria, the discard of 4, leaving 27 explicitly spatial cells out of 101 total) to page 5.

We thank the referee for this comment and apologize for the confusion. There was only one method to classify these cells as spatially modulated by passing the statistical tests (coherence, information, stability) and the information threshold. This is now clarified only in *Materials and Methods* (lines 420-439). In addition, supplementary table 2 shows the number of cells modulated by position with different thresholds for both the statistical tests and the information threshold.

ii) This classification then creates two cell groups, the spatial cells, and the non-spatial cells. For these two cell groups, please provide the statistics of global firing rates (spikes/time for whole trial), on each of the three spatial measures whose combination classifies the cells (in-session stability, spatial coherence, and information rate), and finally waveform characteristics (an amplitude measure, and a spike width measure, for the largest spike on the 4 tetrode tips). For the spatial cells, characterise the maximal peak rates (shown on the top right of rate maps).

All these data sets have been now added to Figure 6.

Less crucial but informative is to divide the trials into two halves, as they have already done for the spatial stability analysis, and compare the integrated firing rate or average spatial rate for, e.g. top 30% peak rate spatial bins for first and second halves of the trial or first vs second trial. I suggest this because in the 6 examples they show of first vs second halves (fig 2), and first and second trial (fig 4), in all 6, the second half/trial shows lower firing rates. Was this a trend?

The referee is correct, this is indeed needed. We added the requested analysis to Figure 6H.

iii) Show more examples of histology with regional labelling (explain abbreviations), with atlas co-ordinates or other co-ordinate system. The region in Figure 1B is somewhat underdetermined. Can the region of the cells be described with any more precision, e.g. more posterior/anterior or ventral/dorsal portions of the central telencephalon? This is doubly important since the goldfish brain is not widely known. In the rodent literature, in describing a new cell type, it is customary to provide a summary figure showing where the spatial cells were recorded. This could be done using line drawings rather than histology photos.

We agree with the referee about the importance of this information. As now mentioned in the last paragraph of the Introduction, the division of the goldfish telencephalon is indeed controversial in terms of boundaries and functionality, and therefore cannot be described with high precision. Nevertheless, we have decided to make the anatomical information concrete, to use as the referee suggested using line drawing adopted from Northcutt 2006 paper on the goldfish brain. In addition, we added information on the whole brain and the section position in the ventral/dorsal axis where we inserted the electrodes. This is presented in Supplementary Figure 1 with additional examples of recording sites. The abbreviations are also explained in the caption to Supplementary Figure 1.

Later part of Results:

iv) Can they provide some analysis, even if not comprehensive, on the directionality of firing. Judging from Fig3 the firing is broadly omnidirectional, ie. taking Fig2A-D cell as an example where the 'neuron was mainly active when the fish was near the left wall of the tank' (p7) irrespective of the path direction taken by the fish near the left wall.

We agree with the referee that an analysis of directionality is needed. For this purpose, we added an analysis (see Figure 4) showing how the allocentric swimming direction of the fish affects the firing rate of the boundary vector cells.

B) Methods

When was recording done relative to the experience of the environments?

After a week or two of training sessions, the implant surgery and subsequent recording sessions were carried out. This is now clarified in *Results* (lines 62-78).

Geometric change manipulation (inserting the Perspex step)

a) This manipulation is surprisingly under-presented.
b) The hypothesis testing is unclear.

Under-presentation.   1) There is no statistical analysis of the (sub)group of cells (n = ?) examined in this manipulation; 2) The authors show data for only n = 2 cells; 3) Moreover both these cells have low firing rates, so if firing is somewhat inhibited, there is not much signal to observe; 4) There is no methods description other than describing the Perspex step itself; when was recording done relative to the experience of the step? First trial, second trial? Etc How long was the trial?

Unclear hypothesis-testing.   The authors should spell out characteristics of firing they expect to see following the manipulation for each hypothesis they are testing. To me, simply put, the cells carry on behaving like boundary vector cells (BVCs), albeit with the noisy firing of low firing rate cells. The cell (figure parts I-P) which fired when it was close to the top of the tank in the baseline trial continues to do so in the manipulation trial, entirely consistently with a BVC whose firing rule is 'fire when close to a boundary in the up direction'. The firing of cell (figure parts A-F) is somewhat less clear because it is quite a low firing rate cell, and the authors autoscale its rate map to the peak in the baseline trial (ie the one with higher rate, see point above). Nevertheless, this cell which fires at the bottom of the tank in baseline continues to fire at the bottom in manipulation, but now also along the top of the Perspex step (as is clearer comparing part B
to E), consistent with a BVC whose firing rule is 'fire when close to a boundary in the down direction'. There is less firing on the top of the step at the very left of the step, true, but this is obscured by autoscaling to the firing rate peak in the baseline trial, and there might also be some novelty-related inhibition of firing around the step. There is also some sign of the waveform amplitude being lower on session 2, so thresholding might be missing some spikes. The overall impression from these two cells is that the BVC model explains their firing pretty well.

We agree with the referee that these data and analyses are needed. Since we have changed the terminology to boundary vector cells, we have replaced these experiments with different control experiments that are better designed to test for boundary vector cells. The new experiments show the effect of the additional boundary on the rate maps of the boundary vector cells (Figure 5 and Supplementary Figure 3).

Regarding the unclear hypothesis testing, the new experiments helped us test the hypothesis that tuning to the boundary would still occur after altering the geometry rather than remaining localized in the same field as in the original setup. This is now clarified in *Materials and Methods* (lines 484-491) and in *Results* (lines 162-179)

Regarding the under-presentation, the new manuscript includes results of 1) n=19 cells tested with this manipulation, out of which 2) n=8 were boundary vector cells. 3) the firing rates were higher for the new cells. 4) The method is now fully described in *Materials and Methods* (lines 484-491).

Beta Oscillation

What is the basis of the 4 db/Hz threshold? Present alternative thresholds and their statistics, or some other way to obviate picking an arbitrary threshold.

We have added an analysis showing that the boundary vector cells are more abundant than other cells over the threshold regardless of the threshold (see Figure 7H and *Materials and Methods* (lines 500-507).

Spatial terminology

Important. The authors are not using the terms principal axis, and the related terms major and minor axes, correctly. In geometry and the disciplines using it (e.g. maths, physics, psychology of space, such as Randy Gallistel, Ken Cheng and others who suggest that to explain re-orientation behaviour in diverse animals such as fish, chicks, and rodents, animals may extract principle axes from their environment), these words have a precise meaning shared across those disciplines. Principle axes are perpendicular to each other and, importantly, pass through the centroid of the body/environment, with the major principal axis being the longest one, and minor axis (axes) being shorter than the major axis. I comment further upon the implications of this misuse of terminology below in the authors characterisation of these cells as 'axial'. It becomes clear as the report proceeds that the authors use the word 'minor axis' to mean any imaginable axis in the environment. For analytical purposes, they test 180 axes. There are not 178 potential minor axes in the standard accepted sense of that term. The authors cannot re-invent these terms' meaning any more than 'hypotenuse' or 'acute angle'. If they mean something else, ok, but then use different words.

We thank the referee for clarifying this important issue. We now refer to the neurons as boundary vector cells as suggested. Specifically, we no longer use the terms "principal axis" or "major/minor axis", and all uses of the word "axis" have been verified for correct context.

Less important. It would help the reader to restrict the different words to apply to the same axis. At different points in the paper, the Z axis is called 'vertical', 'height' and 'depth'. Could the authors just use one verbal label consistently?  It won't be obvious to readers that the Y dimension, 20 cm long in the tank, is called 'length', and the X axis is called width. Given this kind of issue, to help the reader 'fix' the authors' axis label words in mind, it would be helpful to annotate Figure 1A further, using those words, e.g. saying 'Z (height)' and 'X (width)'. The picture should mention the 'Y length' dimension, for clarity, though the Y dimension is in effect collapsed onto the plane.

Less important. When phrases such as 'correlated to a position in the negative X direction' are used, it will be helpful to add an intuitive phrase, like 'i.e. near the left wall of the tank'. Phrases such as 'negative vertical direction' are not that obvious by themselves.

We agree with the referee that there is a need for precise terminology. To make things clear, we now only use the terms "vertical" and "horizontal" to describe the axes of the water tank, with no confusing examples as mentioned here. The axes are now explicitly presented in Figure 1A and Figure 2A.

Axial coding

I found the authors' characterisation of this population in terms of axial coding unhelpful conceptually. Further to the incorrect terminology above, I can't see what the concept is precisely, and what it adds. Its main use as far as I can tell is in opposition to the idea of a Gaussian place field type response. That the cells do not fire like place fields is very convincing but a somewhat obvious 'straw man' hypothesis to undermine.

What is specific to the axial concept? What does the axial concept predict in further studies of these cells, ie how would they behave in such and such an environment or manipulation?
The authors write "Activity was modulated along a preferred principal axis in the environment." "Not all cells were tuned to the major axes of space, but rather to a specified minor axis." And so on.

If the best conceptual way to characterise this cell population is that their firing is axial, one would predict at least some cells firing along the principal axes in the universally-understood sense of that phrase (see above points re 'spatial terminology'.) As mentioned above, proponents of the provocative 'geometric module' theory such as Gallistel and Cheng, whether in its harder or softer forms, have often suggested that to explain re-orientation behaviour in diverse animals such as fish, chicks, and rodents, animals may extract principle axes from their environment. The simplest prediction from such a principal axis view would be that there would be cells firing along the principal axes. However, the number of cells in this report which fire along either of the two principal axes examinable here (Z and X passing through the centroid), is n = 0.  Well that is quite a disconnect.

As the report stands, with the misleading terminology about principal, major and minor axes, casual readers might think the authors have discovered cells which in a straightforward way embody the representations of space suggested by Gallistel. They have not. This is an important issue not to be misleading about, because the rich ideas in the geometric module theory have been very influential. E.g. The initial Ken Cheng paper has >1400 citations, Gallistel's The organization of learning has nearly 5000 citations.

We thank the referee for pointing us to important literature. Indeed, as the referee argues, we need to be cautious, and we should not use a misleading presentation of the data. We do not suggest that our findings show cells which use the principal world axes. Since our terminology was not clear, we no longer use the terms "principal axis" or "major/minor axis", and all uses of the word "axis" have been confirmed for accuracy in the right context. We agree with the referee that the best conceptual way to describe our results is by boundary vector cells.

2) Rather, in both the main and supplementary figures, every single cell can be concisely described as firing at a particular distance and direction to boundaries in the tank, i.e. the walls, bottom and top (water/air surface), including all the cells that fire near corners (see BVCs in Lever et al, J Neuroscience, 2009, Figure 3). Thus, it seems that the Occam's razor view is that this cell population is best described as a boundary vector cell population.

We agree with the referee. We now describe the cells as boundary vector cells.

3) As I have mentioned, the firing of their cell type looks like BVCs in the rodent subiculum. Another cell type in the rodent subiculum is the 'axis of travel' cell as in the (Olson et al, Nature Neuroscience, 2017) report, which in passing also finds about 21% of subiculum cells are boundary vector cells. Importantly, these rodent axis of travel cells were characterised in environments with symmetrical linear tracks. An axis of travel might fire say along half a north-south oriented track on the left of the environment, and fire along the same portion, with mirror symmetry, of the north-south oriented track on the right of the environment. Importantly, these axial travel cells lose their 'axis of travel' tuning in open field environments (ie 2D versions of the 3D open field fish tank used here.) Clearly, these goldfish cells are definitely not like the subicular axial cells, and it would be helpful not to accidentally mislead readers on this point.

We agree with the referee's comment which is in line with the other referees' suggestions. We now describe the cells as boundary vector cells.

References
A golden rule as a referee is not to mention something in the second round that was not mentioned in the first round unless it relates to newly supplied information. Since the references were incorrectly numbered throughout the entire paper, it was not possible to assess some claims and arguments properly without very time-consuming guessing, which could turn out wrong. Accordingly, once the references are corrected, there may be further, hopefully minor tweaks, to address.

We apologize for the inconvenience caused in the initial submission. This has now been corrected. The only reference added deals with how boundary vector cells were hypothesized (Barry, 2006).
