# Supplementary material for: Boundary vector cells in the goldfish central telencephalon encode spatial information
Source: PLoS Biol. 2023 Apr 25;21(4):e3001747. doi: 10.1371/journal.pbio.3001747 (PMC10128963; doi:10.1371/journal.pbio.3001747)
Supplement: Attachment — Submitted filename: Cohen2023_Response to Editor and Reviewers Comments.docx [file pbio.3001747.s014.docx]

Comments by the editor or referees are depicted in black font, our response in red.

**Comments by the editor**

***When revising this work, we also ask that you consider a slight title change to include some functional information in the title, to help our broad readership more easily grasp the significance of this work. Please consider something like:

Boundary vector cells in the central telencephalon of freely swimming goldfish enable navigation

OR

Boundary vector cells in the goldfish central telencephalon encode spatial information

We choose the second option as it better describes our findings.

NOTE: If Supporting Information files are included with your article, note that these are not copyedited and will be published as they are submitted. Please ensure that these files are legible and of high quality (at least 300 dpi) in an easily accessible file format. For this reason, please be aware that any references listed in an SI file will not be indexed. For more information, see our Supporting Information guidelines:

<https://journals.plos.org/plosbiology/s/supporting-information>

The supporting information is in the required resolution. All references in the SI are also cited in the main text.

*Protocols deposition*

To enhance the reproducibility of your results, we recommend that if applicable you deposit your laboratory protocols in protocols.io, where a protocol can be assigned its own identifier (DOI) such that it can be cited independently in the future. Additionally, PLOS ONE offers an option for publishing peer-reviewed Lab Protocol articles, which describe protocols hosted on protocols.io. Read more information on sharing protocols at <https://plos.org/protocols?utm_medium=editorial-email&utm_source=authorletters&utm_campaign=protocols>

All the protocol details were already published in two methods papers which are cited in the text. All required information for reproducing our study is available.

DATA POLICY:

You may be aware of the PLOS Data Policy, which requires that all data be made available without restriction: <http://journals.plos.org/plosbiology/s/data-availability>. For more information, please also see this editorial: <http://dx.doi.org/10.1371/journal.pbio.1001797>

Note that we do not require all raw data. Rather, we ask that all individual quantitative observations that underlie the data summarized in the figures and results of your paper be made available.

All data is available in github with the following link:

<https://github.com/learco1510/BVC_Goldfish.git>

DOI: 10.5281/zenodo.7408609

1) Please provide this summary data in one of the following forms:

a) Supplementary files (e.g., excel). Please ensure that all data files are uploaded as 'Supporting Information'.

b) Deposition in a publicly available repository. Please also provide the accession code or a reviewer link so that we may view your data before publication.

We choose (b) option. The link above provides this access, and it is open for anyone with the link. The link is cited in the main text.

Regardless of the method selected, please ensure your files are invariably referred to (in the manuscript, figure legends, and the Description field when uploading your files) using the following format verbatim: S1 Data, S2 Data, etc. Multiple panels of a single or even several figures can be included as multiple sheets in one (e.g. excel) file that is saved using exactly the following convention: S1_Data.xlsx (using an underscore)

Each figure's data file was uploaded to github and named as suggested above using the figures name. The corresponding indices of all panels in the data file structures are fully described in a ReadMe text file and detailed in an Excel file in the git repository named "data indices.xls".

2) Please ensure that you provide the individual numerical values that underlie the summary data displayed in the following figure panels as they are essential for readers to assess your analysis and to reproduce it:

Fig 1D,E; Fig2-7 all panels

Supplemental Fig2 all; Fig 3C-J; Fig4-7 all panels

Done.

NOTE: the numerical data provided should include all replicates AND the way in which the plotted mean and errors were derived (it should not present only the mean/average values).

All the data is available including all replicates. The way in which the mean and error were derived are mentioned in the caption of the relevant figures.

3) Please also ensure that figure legends in your manuscript include information on where the underlying data can be found (e.g. “The underlying data supporting Fig X, panel Y can be found in file Z.”)., and ensure your supplemental data file/s has a legend.

Done.

4) Please ensure that your Data Statement in the submission system accurately describes where your data can be found.

Done.

**Comments by the referees**

Reviewer #1: The authors have satisfactorily responded to my comments and suggestions. I have no further requests

Reviewer #2: The authors have addressed all my concerns.

Reviewer #3: SECOND ROUND
The manuscript is greatly improved from the initial submission, and close to publication ready.

Statistics
The authors do not provide a summary or the statistics for many of the analyses which are currently just shown as scatterplots/graphs. This is true of all of Figure 6C-H. I am not going to list all, but please attend to this. For instance, in response to this Reviewer's suggestion that, for the boundary cells, the second half of the trial may have reliably lower rates than the first half, the authors present Figure 6H. I asked "Was this a trend?". To answer should be straightforward since this data seems easily amenable to a standard paired t-test, which I imagine would suggest this trend is statistically significant. Can the authors suggest why this might be true, if so?

We added the summary of the statistics to the population analysis panels (Figure 6). The paired t-test suggested by the referee have yielded p=0.011, suggesting the firing rate in the receptive field is somewhat decreasing overtime. This might be a result of an adaptation process to the environment, but further study is needed to clarify this point. This is now clarified in the text (Results, lines 222-227).

Directional tuning
The main issue which needs to be addressed is the swimming directional analysis, The results are in Lines 149-163.
Their methods are in lines 470-484, which I quote in full here:
"Allocentric swimming direction tuning analysis. To test for the effect of allocentric swimming direction on boundary vector cells, we calculated a modulation ratio index; namely, the ratio that minimized the least square error between each cell's two tuning curves - firing rate vs. distance from the preferred boundary while swimming towards it and while swimming away from it (e.g., blue and yellow curves in Figure 4B, respectively). For this purpose, we only use the data recorded when the displacement of the fish was in the range Θ ± 45 and in the range Θ + 180 ± 45, respectively.
To estimate the relationship between the directional and spatial information in boundary vector cells (as shown in Figure 4J), we repeated the process described in (12) to calculate the mutual information between firing rate and location and compared it to the mutual information between firing rate and the allocentric swimming direction of the fish. As in (12), we corrected the inhomogeneous sampling using exactly 48 bins (7.5 degrees each) to calculate the directional information and 48 bins on average for the spatial information."

The authors produce two results:
1) the modulation ratio for 90 degree range centred on theta max and theta+180. That is, the authors have omitted about half of the data. The directions the authors have chosen to compare are arguably exactly the wrong ones to compare. This is due to the behavioural confound with speed and location. Although goldfish and rats are not entirely similar, there is a classic problem of behavioural dependencies, well known in rodent spatial analysis (Burgess et al, 2005, Hippocampus). Notably a head at the front of a body cannot (easily) be facing/moving away from the wall/corner, while that head is simultaneously directly abutting the wall/corner. It is also typically impossible/difficult to do this while travelling at some speed. The expectation is that you can travel at speed right into a corner, but not away from it. Such dependencies would be much less pronounced in a spheroid tank.

We thank the referee for raising these points. We agree that it is hard to differentiate speed and position, and further study in different environments might make this point stronger. Nevertheless, we still see the effect of allocentric swimming direction in the data.

To address the referee comments, we corrected the data bisection for the modulation ratio analysis (Figure 4 B, E, H, K and M) to include all the data (180-degree range centered in theta max and theta+180). As a result, the rate modulation values slightly decreased, but they are still high for some of the examples presented.

As for the point about speed being a possible confound, we analyzed the distribution of swimming direction along distance from the preferred boundary while swimming towards it and away from it (Figure 4 panels C, F, I and L). As can be seen in the new panels, the speed does not change much between the two conditions. Therefore, the speed does not have profound effect on the rate modulations between towards and away from the preferred boundary.

2) The mutual information results show that the amount of locational information carried by the goldfish boundary cells is on average 5x the amount of their directional information. As the authors point out, this is also what Ref 12 found, ie Lever et al, 2009, where the ref 12 claim was that directionality of firing was minimal.
There is an apparent contradiction/paradox because both studies show remarkably similar mutual information results (both studies: Locational info = 5x Directional info) but this goldfish study is claiming, on the basis of the modulation ratio index, that the cells are strongly directional. Furthermore, they claim in Discussion that this is a difference from the rodent BVCs. They are not yet convincing on that point.

The referee is of course correct in all these points. However, it is important to note that only part of the population is direction selective with respect to the preferred boundary (about half of them, depending on threshold, figure 4M). The fact that the population analysis reveals that the locational information is about 5x directional information does not directly relate to the modulation ratio index. The amount of information present in a kinematic variable, position and direction in our case, depends on number of properties. This includes number of degrees of freedom (2 for position, 1 for direction) and noise in this variable. Furthermore, as mentioned by the referee, fish and rats are different animals navigating in very different environments with a different set of sensory cues. e.g., a fish might know its displacement direction in the vertical water column using the derivative sign of hydrostatic pressure, rather than using an internal compass. Altogether, we think the relationship between how directional selective a cell is and the ratio between its information values might differ between systems, and we believe it is only apparent paradox as the referee suggested and not a real one.

Having said that, we did rephrase this part in the discussion and no longer claim for “significant differences between fish and rats”, or “strongly modulations in fish by allocentric swimming direction”. We rather suggest there is an interesting phenomenon here worth looking at in the future.

All the above is now clarified in the text (Results, lines 137-171, Discussion, lines 321-333, Materials and Methods, lines 493-512).

Methods Differences in the studies

A) For directionality analysis, Ref 12, which like this study mainly used a rectilinear-walled box, looked at a subset of BVCs cells (60% of sample) which were also sampled in cylinders. This was because a rectilinear-walled box elicits much more behavioural dependency of the kind referred to above (head, speed, location) than cylinders. Here, the authors have the rectangular box only.

We thank the referee for raising this point. We now mention in the text (Discussion, lines 327-333) that additional study in cylinders might lead to further insight on this issue.

B) The authors have not fully understood the procedure for correction of the inhomogeneous sampling in Study ref 12. They have correctly applied the same number of bins for the directional information and the locational/spatial information. That is an important equivalence. However, what ref 12 referred to regarding correcting inhomogeneous sampling was an additional analysis tool (the so-called PxD maximum likelihood algorithm in Burgess et al, 2005, Hippocampus), trying to correct for (minimise) behavioural dependencies. The authors should remove the claim they are correcting for inhomogeneous sampling with respect to behaviour.

We removed the claim of correcting for inhomogeneous sampling with respect to behavior.

So what is the solution?

The authors are free to conduct more sophisticated analysis, if they want. Supplementary Figure 5 contains a few plots which look at the speed and direction distribution, but this does not directly address the confounds. If the authors want to make the paper acceptable quickly, the minimal-work revision to make is simply to remove the modulation ratio analysis, which is at this point too simplistic. If they want to continue to argue the point strongly that they make in Figure 4, then they need to present an analysis of the firing fields which says that the swimming speeds towards the boundary are the same as those away from the boundary, within that firing field.  There is also an issue if the fields are much bigger for one direction. Thus overall, it might be simplest to remove the modulation ratio, which is only based on some of the data, so not equivalent to the spatial information approach.

As stated above, we added the speed distributions for the two sets of data which includes now all the data (away and towards, Figure 4 panels C, F, I and L), as suggested by the referee. These panels show mean $\pm$ sd of the speed with different distances. We think this data, which includes the swimming speed distribution in the firing fields, addresses the concerns maid by the referee, and is sufficient for the published data to address this point.

Nevertheless, we show here additional analysis- a full speed-map in the water tank, such as in SuppFig5, but bisected into towards and away from the preferred boundary. Same color code is used for each set of maps. Firing fields contours are marked by black solid line for easier assessment. As can be seen, swimming speed barely changes within the firing fields.

This analysis is too cumbersome, and we decided to leave it out of the manuscript.

| Corresponding Cell | **Towards** | **Away** |
| --- | --- | --- |
| Cell 1 (Figure 4A) |  |  |
| Cell 2 (Figure 4D) |  |  |
| Cell 3 (Figure 4G) |  |  |
| Cell 4 (Figure 4J) |  |  |

Histology in Supplementary Figure 1

Please magnify, and provide some regional markers/labels on, the brain slice photo examples shown in this figure. Less important but useful: it would be simpler if the brain slices were in the same orientation as the brain maps. It is awkward for readers to do a mirror translation in their heads.

All histology examples (both in Figure 1B and Supplementary Figure 1) were enlarged, regions and labels were added as suggested by the referee. The mirror orientation is common in neuroanatomy literature (e.g., Northcutt, 2006).

Supp Fig 5

Part D is simply a repeat of part A. This is confusing. Just alter the legend to say that Part A applies to other cells too.

There should be a key displayed for the directions, so we know what 0, -3 and +3 refer to in terms of the actual fish tank.

Supplementary Figure 5 and its legend were revised as suggested by the referee.
