# Supplementary material for: Boundary vector cells in the goldfish central telencephalon encode spatial information
Source: PLoS Biol. 2023 Apr 25;21(4):e3001747. doi: 10.1371/journal.pbio.3001747 (PMC10128963; doi:10.1371/journal.pbio.3001747)
Supplement: Attachment — Submitted filename: Cohen2023_Response to reviewers.docx [file pbio.3001747.s015.docx]

Reviewer #3 remarks:

This is an excellent set of findings, whose report still contains a few kinks, including errors.

We thank the referee for the time and for the detailed comments. We have addressed all the issues raised by the referee here as mentioned below.

Figures
1) Directional scheme icon

I mentioned this before but the authors have not yet placed a circle direction icon indicating what is 0, 90, 180, 270 degrees in their scheme. Fig 1 is best for this. They are adopting zero is Camera East, and 90 is north (anti clockwise progression), like physicists/maths oriented workers. Note many assume that Zero is North and progression is clockwise (90 East), so the directional scheme needs to be clarified for readers in Figure 1 and ideally repeated in Supp fig 2 to appreciate the subsequent figures.

This is indeed an important point. The suggested icon was added to Figure 1 and is now mentioned in the figure legend. We also added the panel as suggested by the referee to Supp Fig 2A.

2) Supp Fig 2

Looking at directions and distances more carefully in Supp Fig 2, I note that the firing rates in Supp Fig 2 are INCREASING away from the boundary, unlike in Fig 4B. The rate distance plots are surely the wrong way round. Presumably the authors need to do a mirror horizontal reflection to make them conform to Fig 4B. These should be decreasing away from the boundary as per the 4B.

We thank the referee for noticing that. Indeed, a mirror reflection was needed. It is fixed in the revised manuscript.

They should note somewhere in the Discussion that not ALL cells fire maximally at the boundary and decrease away, but some show peak firing at a short distance away from it. I note this is true for 5 of the 22 cells shown in sup fig 2.

Added (*Discussion*, line 313-315)

Statistics
1) Statistics and summarising fundings

The authors wrote in the previous revision "In addition, the strength of spatial modulation could not be estimated by either the mean firing rate during the entire recording session (Figure 6F) or…". They are now writing in the latest version that there is a correlation "In addition, the strength of the spatial modulation was also correlated with the mean firing rate during the entire recording session (Figure 6F)." Inspection of the figure shows that this correlation is r = -0.33. That is quite a change from saying there was no correlation.

A key takeaway is that doing statistical tests provides a useful reality-check upon impressionistic summary.

The referee is correct. We are thankful for raising it up in the previous round.

2) With the above caution re impressionistic summary in mind, the summary claims re swimming towards and away from the boundary are not sufficiently supported by the Figure parts 4CFLI. Note that for 3 of the 4 cells, the speed is indeed slower going away for the first (1 cell) and first and second (2 cells) closest distances, which is what matters. Equivalent speeds far away from the boundary eg say 50cm are irrelevant, since the cells do not fire far away from the boundary. It looks to me like speed could indeed be contributing to this effect. At any rate, one cannot make a claim based on 4 cells alone, when the data in those 4 cells is not that compelling, and the 4 cells are a small % of the n = 35 dataset.

We agree with the referee that statistical analysis was needed to further assess the effect of swimming speed in the receptive field of each cell on the firing rate. To do that, we conducted an ANOVA test for the 35 boundary vector cells with direction of swimming (towards vs. away) and distance (10 cm or 20 cm) from the boundary as independent variables and speed as the dependent variable, and we found that for most cells the swimming speed effect was small. We also made sure our speed samples are not time correlated to better estimate the parameters of the speed distributions in Figure 4 panels C, F, L and I.

All the above is now mentioned in the revised manuscript (*Results*, lines 164-174, and *Materials and methods*, lines 517-535).

As an additional measure, we tested the relationship between the mean speed ratio (towards / away) in the receptive field and compared it with the modulation ratio index of each boundary vector cells. We found these measures are not correlated (Pearson correlation test: r=0.02, p=0.88). The results are only presented here because we think it complicates more than contributes for the clarity of the manuscript.

Last, we agree with the referee that further work is needed to establish our claim that the cells are modulated by swimming direction. Therefore, we rephrased through the text the relevant parts. We now only suggest this is an interesting possibility that needs further experiments and analysis in the future (*Results*, lines 171-174, and *Discussion*, lines 334-345).

Supplementary figure 5 simply doesn't address any of these points.
Thus the claim in lines 235-238 needs to be toned down, with more caveat phrasing, and methods mentions should make clear that the analysis has only been done on 4 cells: lines 503-6 implies a generalised analysis.

The referee is right that supplementary figure 5 does not connect to these points. This analysis was done for another purpose. Specifically, we wanted to show that the spatial modulation of the cells (e.g. heatmaps in Figure 2 B,F,J and N) is indeed a result of position encoding rather than other aspects of navigation. For example, if the distribution in space of the fish swimming speed had a gradual pattern, that alone could explain the observed phenomenon. This part was rephrased to make it clearer (now lines 246-251)

Regarding the methods section, as mentioned above, we now did a generalized analysis, and the methods section of the swimming direction analysis was changed accordingly (*Materials and Methods,* lines 517-521)

3) Please report statistics with more detail.

a) I leave this to journal discretion, but I would favour more stats description and values appearing in the main text, and not having to look at the figures all the time. It helps give a sense of what are stronger and weaker effects, and helps the reader understand exactly of what the statistical test is composed. See b and c below.

Statistics were added to the text (*Results*, lines 216-240).

b) It should be clear in Figure 6 that 6H is not a correlation test, but a t-test. Since a scatterplot is shown, in a context of Pearson correlations, that could mislead. The values are likely correlated, but that is not the analysis done. Report t value, df. Same goes for 6G.

We added t-values and df to Figure 6 H and G and are now mentioned in the text (*Results,* lines 229-235).

c) I am unclear re precisely what on the Y axis is being correlated in 6CDEF. It looks like, and should be, the spatial tuning operationalised as the max-p value. (This helps to understand why the correlations are negative.) However, the Y axis in these 4 scatterplots is labelled as Cell hash, ie an ID number. Not sure what is happening here. Are the authors converting max-p values into a rank? If so, Pearson's correlation is not the right test. If not, relabel the Y axis appropriately.

Correlations were indeed calculated with the max-p values and therefore were negative. Y axis was previously labelled as the cell ID (sorted by descending max-p value) solely for easier graphical assessment of the scatter plot (with a max-p axis many points would be around 0),

For the correlations to correspond to the graphics, and for the graphics to stay clear, we relabeled the y-axis as rank-ordered max-p, and correlations were changed from Pearson to rank-order correlation.

4) 6I is incorrectly using a linear Pearson's correlation, where it should be a circular-linear one. I am not disputing there is likely no correlation, but the authors must understand that a linear correlation test based on the Y axis having 360 at the top, and zero at the bottom is not appropriate. 350 at top is very close to 10 degrees at bottom in reality, and so forth. Best would be to show this plot in the 6B circular format.

The referee is correct. The suggested figure 6I replaced the previous one, statistical test was changed accordingly to the one suggested by the referee (*Results*, lines 238-240).

Modulation ratio

Using the full data plus or minus 90 not 45 is a nice improvement. I note the mean modulation ratio has dropped accordingly. Two points remain.

a) Add modulation ratio values to the plots in Fig 4, so readers have intuition for this measure.

Done.

b) Relatedly, importantly, explain if the modulation ratio is signed or unsigned. Figure 4M shows that ALL the modulation ratio values are ABOVE unity. The Methods do not make it clear whether the ratio is defined as always BLUE/TOWARDS divided by YELLOW/AWAY, or rather TOWARDS/AWAY or AWAY/TOWARDS. There is quite a difference between these possibilities. If the ratio is BLUE/TOWARDS divided by YELLOW/AWAY, then ALL the cells fire at a greater rate towards the boundary.

This is unsigned for easier assessment of the effect size. This is now mentioned in the text (Results, line 156-159, and Materials *and Methods,* lines 515-516).
